# Design and validation of a bioethical assessment instrument for public health policies involving behavioral change: A mixed-methods study^☆^

**DOI:** 10.1016/j.puhip.2026.100742

**Published:** 2026-02-09

**Authors:** A. Castillo Martínez, P. Aristizábal Castrillón, L.J. Lopez Erazo, M. Montoya Villegas, L.A. Tamayo, M. Montagut Ascanio, J.P. Borda, E.F. Lasso

**Affiliations:** a Medicine and Surgery Universidad Nacional de Colombia, Psychiatry Universidad Nacional de Colombia, Colombia; b Pontificia Universidad Javeriana in Bogotá, Colombia; c Medicine and Surgery Universidad del Valle, Colombia; d Pontificia Universidad Javeriana in Cali, Colombia; e Universidad Nacional de Colombia. Master in Interculturality, Development and Territorial Peace from the Institute of Intercultural Studies of the Pontificia Universidad Javeriana, Colombia; f Medicine Universidad Autónoma de Bucaramanga, MBA Healthcare from Strayer University, USA; g Pontificia Universidad Javeriana Bogota, Master of Public Health Candidate with Emphasis on Mental Health from King's College London, UK; h Medicine and Surgery Universidad del Cauca Psychiatry Universidad del Valle Specialty in University Education Universidad IcesiBioethics Universidad El Bosque, Colombia

**Keywords:** Health policy, Bioethical issues, Validation study

## Abstract

**Objectives:**

Growing interest in developing bioethical evaluation models for public health, together with limited consensus on fundamental moral values, highlights the need for a framework to guide ethical decision-making in this field. The Nuffield model has been proposed as an alternative for addressing ethical conflicts at the population level, as it offers a more suitable approach to the complexities of behavioral-change interventions than traditional clinical bioethics. Consequently, methodological tools are needed to guide decision-makers. This study sought to design and validate an instrument for the ethical evaluation of public health policies involving behavioral change.

**Study design:**

Scale development and validation content study.

**Methods:**

Reflective equilibrium was used as the overarching methodological strategy for instrument development. A three-phase mixed-methods (qualitative–quantitative) process was conducted, which included semi-structured interviews and expert validation using the Delphi technique.

**Results:**

We developed an instrument for the bioethical evaluation of public health policies that include a behavioral-change component, comprising nine domains and 32 items. Two Delphi rounds were carried out for expert validation, followed by a pilot test that informed adjustments to the final version of the instrument.

**Conclusions:**

The methodological approach enabled the construction of the instrument by contrasting and ensuring coherence among predefined domains and the theoretical frameworks analyzed. Through the application of reflective equilibrium, new domains, integrity, participation, and interculturality, emerged in addition to those identified in the literature, resulting in an instrument with a strong theoretical foundation and expert validation.

## 1 What this study adds

- A new instrument for the ethical evaluation of public health policies that specifically involve changes in behavior.
- An instrument designed to evaluate interculturality in order to develop solidarity-based strategies that fairly recognize and include identity characteristics in public policies.
- An evaluation of coercion in public policies using the scale proposed by the Nuffield management model, enhanced with level-specific questions to enable gradation and promote ethical reflection.

## 2 Implications for policy and practice

- The instrument promotes ethically grounded decision-making in public health policies involving behavioral change.
- It incorporates context-sensitive domains like interculturality and integrity, making it relevant for diverse and inequity-prone settings.
- It strengthens accountability and stakeholder participation, enhancing legitimacy and trust in health policy processes.

## 3 Introduction

Public health bioethics is a relatively new field that faces difficulties in the application of the principles of the classical model of clinical bioethics. The model based on principles of Beauchamp and Childress [1], although widely used in clinical settings, is insufficient to address bioethical issues in public health and in Public Policy (PP) design [2]. The principalist framework is primarily oriented toward individual clinical encounters and therefore privileges considerations such as respect for autonomy through individual informed consent. In contrast, the scope of public health interventions is inherently population-based—even though some interventions, such as vaccination, may still require individual consent. As a result, the ethical analysis of public health actions demands a different set of discussions and considerations, many of which are closely linked to political philosophy. Developing an appropriate model for the ethical evaluation of public health policies is essential, as it can help prevent the formulation of policies that conflict with the principles required for social cooperation in democratic contexts.

In the literature, several models and instruments for bioethical analysis and evaluation of public health programs are identified [3,4], in which values such as non-maleficence, beneficence, justice, scientific validity, autonomy, communication and transparency [1] stand out. However, there is no clear consensus on the fundamental moral values in public health, which underlines the need to build a reference framework to guide ethical decisions in this field [5]. In response, the Nuffield Council in the United Kingdom brought together international experts from various disciplines to develop values and operational principles to guide decision-making in public health [2], especially those that incorporate a component of behavioral change, from an ethical perspective.

The Nuffield model provides an ethical foundation adaptable to today's complex public health challenges. It balances public health with individual freedom: it allows coercion when an individual's actions affect public health, provided that there is proportionality and that certain criteria for public cooperation between citizens considered free and equal are respected [6]. To this end, the model incorporates an instrument that balances the promotion of healthy behaviors, according to public interest, respect for the freedoms and rights of citizens and the search for social justice [[7], [8], [9]]. It seeks to protect vulnerable populations and respond to social inequalities [2,10].

The Nuffield model acts as an input to discuss the core values for ethical evaluation of public health policies in Colombia, since it is in line with the priorities set out in the 1991 Constitution. By incorporating a participatory and pluralistic dimension and aligning with public health policies in values such as legitimacy, political participation, justice, equity, scientific evidence and transparency [2], which have been in the forefront in the public discussion in Colombia.

In Colombia, there has recently been an increasing interest in the ethical discussion of public health policies that involve changes in behavior, without there being a consensus on the values that should guide the ethical evaluation of such policies. In 2010, UNESCO has promoted the creation of committees like the National Council of Bioethics (CNB) in Colombia to involve citizens in the evaluation of the effects of scientific advances on their lives, as well as in the analysis of ethical tensions in public policies [11]. In addition, the OECD has proposed the BASIC Toolkit, a set of instruments designed to guide the development of public policies grounded in behavioral analysis. Its aim is to anticipate the effectiveness of interventions and support the formulation of more robust policies. The toolkit also provides ethical guidance at each stage of policy design, aligned with bioethical frameworks such as that of the Nuffield Council on Bioethics [12].

In 2014, the National Planning Department (DNP) constituted the National Assessment System of Management and Results, in support of the national effort to strengthen public administration and in to structure the conceptual bases and processes to evaluate and follow-up the implementation of public policies in Colombia. However, this system has no specific interest in the ethical dimension of public policies [13].

Few publications were found in Latin America and Colombia related to bioethical analysis of public health policies that involve behavioral change. In Colombia, publications related to bioethics and public health have been limited to the compilation of international theoretical frameworks and to the discussion on the importance of adopting a multicultural approach to health care, and on the role of the State in regulating the health system market to promote access and comprehensiveness in care [14]. Proposals for methodologies or tools to guide decision-makers are needed.

The objective of this study is to design and validate an instrument for the ethical evaluation of public health policies that involve changes in behavior, based on the discussion about recognized models, such as Nuffield, and relevant perspectives for the Latin American context.

## 4 Methods

The construction of the instrument was inspired by the method of reflective equilibrium, one of the most influential Coherentist strategies for the justification of normative principles in contemporary philosophy, and widely recognized in applied ethics, bioethics, and epistemology [15,16]. Thus, the domains of the instrument emerged from the “*back and forth*” contrast and review, to achieve a coherent system of the following elements: (a) the conclusions derived from the hermeneutic analysis, an interpretive method that enables understanding the meaning of texts or experiences within their contextual background, of key theoretical frameworks, notably the Nuffield model and the OECD's BASIC Toolkit; (b) discussions within the research team; (c) the perspectives of experts in public health policy; (d) the formulation of normative criteria (instrument domains) based on the articulation of positions derived from the previous levels; (e) the results of the pilot test in which the instrument was applied to a specific public policy; and (f) an expert feedback process designed to ensure the external coherence of the final instrument.

A mixed methodology exploratory sequential (qualitative - quantitative) was used in three phases:

*First phase*: A mapping of potential participants was conducted, identifying individuals with experience in the design, implementation, or evaluation of public policies, including experts at local and national levels and decision makers from various institutional or academic sectors. The selection criterion required that all participants be experts in the development or evaluation of public policies or be decision makers in national or local contexts, be over 18 years of age, and meet no additional inclusion or exclusion criteria. In total, 11 participants were identified. A hermeneutic analysis of the literature on existing models for assessing bioethical aspects of public health policies involving behavioral change was conducted to inform the development of the instrument's domains. Based on the findings of the literature review and the hermeneutic analysis, a semi-structured interview guide was constructed.

*Second phase:* In-depth interviews were conducted with the stakeholders identified in the mapping process. After obtaining informed consent, interviews were carried out to explore participants’ understanding and perspectives, following a phenomenological approach aimed at capturing their lived experience and its contextual meaning. Interview recordings were transcribed verbatim, and a thematic analysis was performed using an open, inductive coding process. The verbatim data were exported to ATLAS. ti for analysis. The research team received training to minimize interviewer bias. A total of 10 interviews were conducted, 2 in person and 8 virtually, with an average duration of approximately 180 min, and data saturation was achieved. One of the researchers conducted the initial analysis by familiarizing themselves with the data and subsequently applying inductive coding. This process led to the development of the following categories and subcategories: legitimacy, transparency, participation, intersectorality, interculturality, evidence, equity, and, finally, challenges and opportunities regarding the usefulness of an instrument designed to evaluate the bioethical aspects of public policies.

*Third phase:* With the revised version of the instrument, an expert validation process was conducted through two iterations using the Delphi method [17,18], A structured questionnaire was administered to assess item comprehension, ordering, and, particularly, the coherence of the domain structure and the relevance of each domain and its corresponding items. This process aimed to determine whether the instrument adequately evaluated the constructs it was designed to measure. Informed consent was also obtained for this phase.

*Pilot test*: The research team was divided into two subgroups to evaluate Article 5 of Law 2120 of 2021, which establishes the technical regulations for nutritional and front-of-package labeling requirements for packaged foods intended for human consumption in Colombia. This evaluation was conducted using the final version of the instrument, after which the remaining adjustments were incorporated to produce the definitive version.

*Sample Size and Sampling Strategy:* Participants in the second and third phases of the project were selected through purposive sampling based on the previously constructed participant map. Ten of the eleven mapped individuals were interviewed. For the criterion validation, the Delphi methodology was applied; nine experts participated in the first round and seven in the second, consistent with the literature, which indicates that expert panels for this technique typically range between seven and thirty participants.^16,17^The individual initially mapped who did not participate in the semi-structured interview was able to take part in the first round of the Delphi process, ensuring that their perspective was incorporated at least during this stage.

*Information gathering techniques:* The necessary institutional contacts were carried out, and interviews and contacts were defined for the project's second and third phases. Once informed consents were signed by participants, interviews were recorded in the second phase of the project, and then the verbatim data were transcribed and exported to the ATLAS Ti software for analysis.

For the validation part, information was collected with the defined experts and was then evaluated by the research group to define the final version of the instrument. The project was reviewed and approved by the Ethics Committee of the Hospital Universitario Fundación Valle del Lili (ethical reference number 376–2022).

*Data Management and Statistical Analysis:* The ATLAS Ti package was used for the analysis of qualitative data. Visualization tools were built and analyzed: analytical networks, word clouds and cooccurrence tables.

For the quantitative phase involving the Delphi technique, and based on the experts’ responses from both iterations, a database was constructed and analyzed using Stata version 13.0. An exploratory analysis was conducted to identify and correct missing data. Once the accuracy and completeness of the dataset were ensured, a univariate analysis was performed to calculate means and their corresponding standard deviations, as well as medians and interquartile ranges for each item evaluated in each iteration. These analyses were based on a Likert scale ranging from 1 to 7 (1 = complete agreement; 7 = complete disagreement).

## 5 Results

The main result of this research was an instrument for the bioethical evaluation of public health policies with a component of behavioral change (Appendix1).

**Fig. 1:**
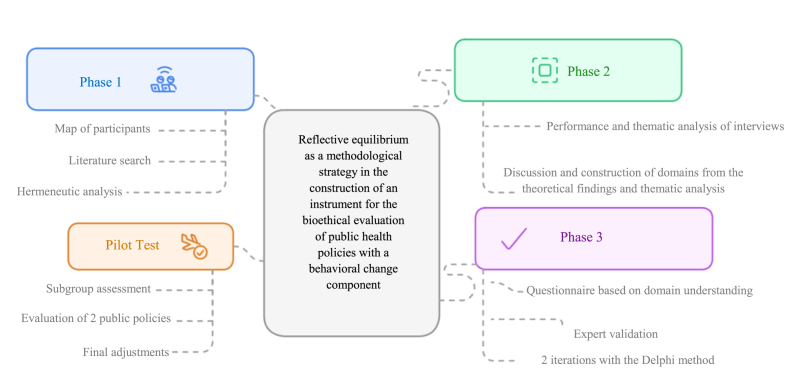
Construction phases of the instrument.

Below is a diagram of the building process of the domains of the instrument (Fig. 1).

### 5.1 Phase 1 results

Participants Map: In the participant-mapping process, ten individuals were characterized, each with broad and diverse experience in key areas related to public health, public policy, and bioethics. Of these participants, ten were experts in the formulation, implementation, and evaluation of public policies, particularly those related to health and social programs for vulnerable populations, including gender-based violence and social exclusion. One participant specialized in the behavioral sciences, with a particular interest in research ethics and bioethics. Seven participants were university professors with experience in public health and bioethics programs, and ten served in government agencies, contributing expertise in areas such as sexual and reproductive health, epidemiology, and gender-sensitive public policies (Appendix 2).

Theoretical rationale and definition of the domains as a result of the literature hermeneutic analysis: As a result of the hermeneutic analysis of the literature, the theoretical basis of the instrument and the definitions of the domains to be integrated in the instrument were obtained [[4], [5], [6], [7], [8],^12^,[19], [20], [21], [22]].

### 5.2 Phase 2 results

The thematic analysis of the in-depth interviews with the different participants characterized in the map aimed to investigate the understanding and insight from the perspective of the participants on the bioethical evaluation of public policies, an analysis used in the development of discussion panels within the research group, to design the instrument, focusing on the construction of a structure of domains that would account for the concept: bioethical evaluation of public polices with a focus on behavioral change (Appendix 3). The following table presents the general structure of the domains resulting from Phase Two; it also displays the items within each domain and the type of questions included in the instrument (Table 1). For additional information on the qualitative findings, see Appendix 4.

**Table 1:** Structure of domains, items, and type of question of the instrument.

Domain	Domain Items	Type of response
Transparency	PP accessibility to citizenship	Likert
	PP Ease of comprehension to citizenship	Likert
	Financing information of the PP	Likert
	Exposure of the benefits, risks, and limits of the PP	Likert
	Education to understand how the PP influences citizenship	Closed (Yes/No)
	Inclusion of some type of PP assessment	Closed
	Disclosure plan for the PP evaluation results	Closed
Evidence	A rigorous balance between the potential risks and benefits of the PP	Likert
	Rigorous review of evidence that considers conditions, perspectives, and contexts of different population groups	Likert
	Clarity to citizens in case of lack of evidence	Likert
Integrity	Articulation of the P.P with constitutional principles and current regulations	Likert
	PP according to legally established democratic processes	Likert
	Identification of conflicts of interest in the PP	Closed
	Written declaration of identified conflicts of interest	Closed
	Legal and ethical strategies to manage conflicts of interest	Likert
Participation	Active participation of diverse population groups in the PP	Likert
	Inclusion of results of the PP participation process	Likert
Equity	Evaluation of the inequities in the distribution of benefits and risks of the PP	Likert
	Strategies to prevent discrimination in the PP	Likert
	Strategies to eliminate discrimination in the PP	Likert
	Differential approach in the PP	Likert
Interculturality	Consideration of cultural and territorial factors in the design and implementation of the PP	Likert
	Consideration of territorial conditions in the design and implementation of the PP	Likert
	Diagnosis with differential approach of health inequalities in the PP	Likert
Social responsibility	Identification of actors involved in the PP	Likert
	Consideration of incentives for actors and ethical evaluation of their impact on the problem under policy	Closed
	Regulations for the actors involved in the problem under policy	Likert
Coercion (Types of coercion) Types of coercion are not mutually exclusive	Monitoring the current situation without coercion in the proposed PP.	Closed
	Educational and informative strategies in PP to face the object of the intervention	Likert
	PP requirement for participation in behavioral change activities or programs	Closed/Likert
	Behavioral change strategies to guide citizens' choices	Likert
	Incentives to channel behavioral changes	Likert
	Disincentives to channel behavioral changes	Likert
	Partial restriction of individual freedoms for the sake of public health	Likert
	Complete restriction of individual freedoms for the sake of public health	Likert/Likert/Closed
Consent	Evaluation of consent by an ethics committee	Closed
	Informed consent from the persons subject to intervention in the PP	Closed
	Assurance of protection, privacy, and confidentiality in the PP of behavioral change	Likert

### 5.3 Phase 3 results

Two iterations of the process were carried out using a questionnaire focused on the understanding of the domains using Delphi technique (Annex 5).^36^

#### 5.3.1 Results from the first and second round

To validate the newly developed instrument, the expert panel evaluated the relevance and clarity of the eight domains and 40 items included in the initial questionnaire. As a research team, we decided to modify or eliminate items with average scores below 75% of the maximum value on the Likert scale, in accordance with the experts' assessments. The response scale ranged from 1 to 7 (1 = completely agree, 4 = neutral, 7 = completely disagree). Following this statistical analysis and considering insights from the qualitative analysis of the experts’ comments, the items that were validated, eliminated, or selected for inclusion in the next validation round were identified (Table 2). Decisions to eliminate or modify items in both validation rounds (Table 3) were based not only on quantitative results but also on qualitative contributions from the expert panel.

**Table 2:** Delphi methodology - first round (n = 11).

	Mean ± SD	IQR (Q1–Q3)
**Question 1**	3.18 ± 1.99	(1.50–4.50)
**Question 2**	2.91 ± 2.51	(1.00–4.50)
**Question 3**	3.18 ± 2.18	(1.50–5.00)
**Question 4**	5.18 ± 2.44	(3.00–7.00)
**Question 5**	4.18 ± 2.18	(2.50–6.00)
**Question 6**	3.82 ± 2.40	(2.00–6.00)
**Question 7**	5.00 ± 2.45	(2.50–7.00)
**Question 8**	3.64 ± 1.96	(2.00–5.50)
**Question 9**	4.45 ± 2.25	(2.50–6.50)
**Question 10**	3.91 ± 2.26	(2.00-5.50)

Note. IQR = Interquartile Range. Consensus threshold = 75% of maximum Likert value (0.75 × 7 = 5.25).

**Table 3:** Delphi methodology - second round (n = 7).

	Mean ± SD	IQR (Q1–Q3)
**Question 1**	2.71 ± 2.36	(1.00–3.50)
**Question 2**	2.71 ± 2.36	(1.00–3.50)
**Question 3**	3.00 ± 2.38	(1.00–4.50)
**Question 4**	4.71 ± 2.36	(3.50–6.50)
**Question 5**	5.29 ± 1.89	(4.50–7.00)
**Question 6**	4.00 ± 2.45	(2.00–6.00)
**Question 7**	4.43 ± 2.70	(2.00–7.00)
**Question 8**	2.86 ± 2.04	(1.00–4.00)
**Question 9**	4.71 ± 2.36	(3.50–6.50)
**Question 10**	5.43 ± 2.70	(4.50–7.00)

Note. IQR = Interquartile Range. Items scoring >5.25 met the consensus threshold.

In the first round of the Delphi process, mean scores for the ten evaluated items ranged from 2.91 to 5.18, with standard deviations between 1.96 and 2.51. None of the items reached the predefined consensus threshold (≥5.25), although two items—related to conceptual clarity and the ethical framing of the domain, approached this criterion (Means: 5.00 and 5.18). In the second round, two items met the consensus threshold (Means: 5.29 and 5.43), indicating improved clarity and relevance following revisions made between rounds. Although the remaining items did not surpass the consensus value, they demonstrated reduced variability and benefitted from qualitative refinements that strengthened the final version of the instrument. These findings helped identify which components required substantial adjustments and which reached an adequate level of expert agreement.

#### 5.3.2 Pilot test

For the pilot phase, the research team was divided into two subgroups to evaluate Article 5 of Law 2120 of 2021, which establishes the technical regulation for nutritional and front-of-package labeling requirements for packaged foods intended for human consumption. This evaluation was conducted using the final version of the instrument. As a result, no items were eliminated from the originally proposed domains; only minor wording adjustments were made to improve the clarity of specific questions.

## 6 Discussion

The methodological approach of the instrument was inspired by reflective balance [15]. This method allowed us to create the instrument through contrast and the search for coherence between the defined domains; the theoretical positions analyzed;^2,318-21,23^ the discussion of the research team (which included members with specialized knowledge in bioethics, public health, evidence-based medicine and political philosophy); the perspectives of experts in public health and bioethics; and the results of a pilot test to evaluate the relevance of the instrument when used in the analysis of specific policies. This approach required the willingness of the research team to review their initial intuitions in light of the emerging elements in the research process. We consider that this methodological strategy is one of the strengths of this research.

The initial set of domains emerged mainly from the literature analysis; from the Nuffield model, the *BASIC toolkit* and the systematic review of 13 models developed by Henein and Ells^.^ [19]. This initial input was reviewed and adjusted. The management model of the *Nuffield Council on Bioethics* mentions that the State must be primarily responsible for safeguarding the health of the population, adopting a management, supervision, and orientation approach, without falling into paternalistic control. It also must commit to reducing health inequalities, with a priority focus on vulnerable groups, although some authors argue that this point could be more explicit^.^ [23]. The model raises the importance of respecting individuality and advocates for solutions with the minimum degree of coercion possible to achieve public health goals, while recognizing that health depends not only on individual decisions, but also on social, economic, cultural, and environmental factors, and that it requires action at the community level.

The BASIC toolkit proposed by the Organization for Economic Co-operation and Development (OECD) was another input for the hermeneutic analysis [12]. This toolkit assumes that context and behavioral biases influence our decision-making [24], therefore, it incorporates the study of these factors in the design and implementation of public policies with a behavioral change approach that are really effective and have a greater receptivity on the part of the citizens. This instrument also promotes ethical reflection in the formulation and evaluation of public policies with a focus on behavioral change.

New domains not considered in the Nuffield nor in the 13 models analyzed by Henein and Ells emerged during the discussion and the application of the reflective equilibrium methodology [19,22,25]. One of these new domains was integrity (5). Although both the Nuffield and other instruments partially address this aspect through conflict-of-interest management, the team decided to expand it due to the emphasis given by the experts during the interviews. They pointed out that one of the main problems in Colombia is how corruption affects decision-making and the design of public policies. For this reason, we believe it is relevant to combine conflicts of interest management with the need to ensure that public policies, which should be aligned with the current regulations of the Colombian context, are framed in an ethical analysis of the circumstances.

Another domain that emerged was that of participation, understood as a plural and effective process in which all stakeholders participate in decision-making. Although this concept is present in Marckmann's proposal [3], the research team decided to give it a greater relevance, based on the results of the analysis of the interviews with experts in phase [2], in order to articulate the principles of public health in the construction of public policies. In this domain the notion of *Accountability for Reasonableness by Daniels,* [16,26] was taken as an additional reference, which underlines the importance of participation to ensure that the reasons behind public policies are indeed justifiable for all stakeholders.

Interculturality is the third emerging domain resulting from a reflection based on Latin American and feminist literature and whose relevance was further reinforced by the experts [21,[27], [28], [29], [30]]. This concept significantly complements the domains of participation and equity, since it deepens in the understanding of social structures based on gender, ethnicity, sexual orientation, abilities, among other aspects. Interculturality as a concept seeks to formulate solidarity strategies to recognize and include identity characteristics fairly into policies [31]. It also invites us to reflect on how different cultures can contribute to the scientific field, promoting an opening that strengthens the critical and expansive spirit of science [32]. We consider that this domain is another novel aspect.

The domain of coercion developed in the instrument is based on the scale proposed by the Nuffield management model [2]. This scale provides clear and concise criteria to identify the level of coercion in a PP. However, the team complemented this scale by incorporating questions to analyze the concept of coercion as a continuum and formulating questions at each level of coercion that facilitate gradation and ethical reflection, based on the consideration that the different degrees of coercion are not mutually exclusive.

One of the limitations identified is that the validation of the instrument was conducted using expert opinion through interviews and the Delphi methodology. However, the nature of the concepts addressed by the instrument does not allow for the development of a purely quantitative scale. In this sense, the instrument is not designed to measure ethical criteria by assigning numerical values. Instead, it provides a qualitative assessment intended to offer a comprehensive ethical perspective on how a public policy (PP) is structured. This assessment is subject to the judgment of the PP committee or expert applying it.

The tool is conceived as a resource to promote reflection and to offer an integrated overview. It is a validated instrument that encourages users to ask critical questions and to consider key ethical principles throughout the formulation, design, and implementation of public policies with an emphasis on behavior change. Although non-probabilistic sampling may introduce bias and limit the generalizability of the findings at the international level, the theoretical framework of this study integrates diverse normative perspectives that aim for universal applicability, consistent with the philosophical models that underpin it. As noted by Lee [20], the Nuffield model [2] offers a plausible alternative in the absence of broad consensus on a conceptual framework for public health bioethics, if achieving such consensus is even possible or desirable.

This study aimed to design and validate an instrument for the ethical evaluation of public health policies involving behavioral change. The result was an instrument comprising nine domains and 32 items. The methodological approach supported the construction of the instrument through contrast and coherence across predefined domains and the theoretical frameworks analyzed. The use of reflective equilibrium led to the identification of additional domains, integrity, participation, and interculturality, beyond those reported in the literature. This process produced an instrument with a strong theoretical foundation and expert validation.

## Author contribution

A. Castillo Martínez: conceptualization, research, methodology, formal analysis, software, visualization, validation, acquisition of funds, writing and original draft of the manuscript, revision and editing of the manuscript.

P. Aristizábal Castrillón: Conceptualization, research, methodology, drafting, original draft of the manuscript, review and edition of the manuscript, acquisition of funds.

L. J Lopez Erazo: analysis of the information, original drafting of the manuscript, review and edition of the manuscript.

M. Montoya Villegas: Formal analysis, original drafting of the manuscript, review and editing of the manuscript.

L.A Tamayo: Research, drafting: results and conclusion, review, and editing.

M Montagut Ascanio: Conceptualization, research, original drafting, and revision.

J.P Borda: Conceptualization, research, acquisition of funds, review and editing of the manuscript.

E.F Lasso: Project Management, funding procurement, conceptualization, research, methodology, formal analysis, review and editing of the manuscript.

## Ethical statement

The study was approved by the Ethics Committee of Fundación Valle del Lili University Hospital.

## Funding

This project was funded through an unrestricted research grant from Tecnoquimicas S.A. to the Bioethics Center of Fundación Valle del Lili Hospital.

## Declaration of competing interest

The authors declare that they have no known competing financial interests or personal relationships that could have appeared to influence the work reported in this paper.
