# Supplementary material for: Design and validation of a bioethical assessment instrument for public health policies involving behavioral change: A mixed-methods study
Source: Public Health Pract (Oxf). 2026 Feb 9;11:100742. doi: 10.1016/j.puhip.2026.100742 (PMC12915271; doi:10.1016/j.puhip.2026.100742)
Supplement: Multimedia component 1 [file mmc1.docx]

**Instrument for bioethical evaluation of public health policies with a behavioral change component**

This instrument guides the bioethical evaluation of public health policies with a behavioral change approach in its design and implementation phases and, in that sense, serves as a guide for decision makers, public health policy-makers and citizen observers. The design of this instrument used, on one hand, the model of the Nuffield Council on Bioethics as a bioethical framework and, on the other, the BASIC toolkit of the OECD as a model of behavioral change approach in public policies. In addition, other bibliographic references were used to contrast these models, especially from a Latin American perspective.

The following instrument was developed based on the discussions of the research team on the models and references mentioned, and the opinion and contribution of the experts.

**Bioethics in public health**

UNESCO defines bioethics as the systematic, pluralistic, and interdisciplinary study of the ethical issues raised by medicine, the life sciences and the social sciences when applied to human beings, and their relationship to the biosphere, which includes issues relating to the availability and accessibility of scientific and technological advances and their applications.

Bioethics is fundamental to understanding health as a cross-cutting value and a right for all society, linked to good living and general well-being. Since health is a right, the State must:

1. Care for and solve basic and important needs of society both individually and collectively
2. Provide conditions that enable people to be healthy while reducing health inequalities. For example, healthy spaces, environmental regulations, and guarantees of public goods and services
3. Bring together collective efforts of different actors to generate and contribute measures to improve health.
4. Allow people to live healthier lives by giving them advice and information.
5. Pay attention to the most vulnerable.

In addition, the State has some restrictions on its role of promoting public health, this means that it must:

- Try not to interfere in the path that each individual chooses to lead a healthy life.
- Minimize interventions that affect individuals’ personal lives.

Below are a series of questions divided into different components, fundamental to the bioethics of public health.

- **Transparency**

The implementation of a public policy is transparent if information regarding its benefits, risks and limits is open and understandable to the public. It is also important as a criterion of transparency; it means it is clear as to how such a policy is to be financed and that the results of the evaluations carried out about it are publicly disclosed. Furthermore, it is essential that it is clear the way the public policy with a behavioral change approach tries to influence citizenship.

*Questions*

- Is the information on the proposed public policy accessible to the public?

| Totally disagree | Disagree | Neither agree nor disagree | Agree | Totally agree |
| --- | --- | --- | --- | --- |

- Is the information on the proposed public policy understandable by the public?

| Totally disagree | Disagree | Neither agree nor disagree | Agree | Totally agree |
| --- | --- | --- | --- | --- |

- Is there sufficient information on how the proposed public policy is to be financed?

| Totally disagree | Disagree | Neither agree nor disagree | Agree | Totally agree |
| --- | --- | --- | --- | --- |

- Are the benefits, risks, and limits of the public policy to be implemented disclosed?

| Totally disagree | Disagree | Neither agree nor disagree | Agree | Totally agree |
| --- | --- | --- | --- | --- |

- Was a pedagogical strategy defined so that citizens understand who and how the public policy is trying to influence them?

| Yes | No |
| --- | --- |

- Does the policy design include any kind of evaluation?

| Yes | No |
| --- | --- |

- Does the policy design include a plan for communicating the results of the evaluation?

| Yes | No |
| --- | --- |

- **Evidence**

One of the fundamental bioethical criteria in the design of a public policy is the evidence on the effectiveness and possible risks and benefits of such a policy. A review of possible evidence bias should also be incorporated. In public health, there are sometimes situations in which sufficient evidence cannot be provided to implement a public health intervention. When this happens, transparency is crucial, and the policy must declare that the risks or benefits cannot be accurately estimated.

*Questions*

- Was a careful balance made between the potential risks and benefits of the intervention?

| Totally disagree | Disagree | Neither agree nor disagree | Agree | Totally agree |
| --- | --- | --- | --- | --- |

- Was there a methodological review of the evidence that covers the conditions, perspectives, and contexts of different population groups?

| Totally disagree | Disagree | Neither agree nor disagree | Agree | Totally agree |
| --- | --- | --- | --- | --- |

- In case of lack of evidence, was this situation made explicit and clear?

| Totally disagree | Disagree | Neither agree nor disagree | Agree | Totally agree |
| --- | --- | --- | --- | --- |

- **Integrity**

Integrity from the public sector is defined by the OECD as adherence to and compliance with norms, principles, and ethical values to prioritize public interests. A process of designing and implementing public policies in a democratic government with integrity assumes and implements the necessary instruments and processes to identify conflicts of interest, in order to resolve them in accordance with the legal framework and ethical principles. An process with integrity in a democratic government is one that respects human rights within the corresponding constitutional framework, serves the general interest, respects the rule of law and impartiality in the application of the rules, seeks efficiency and the correct use of public goods, seeks transparency and is willing to be held accountable.

*Questions*

- Are the reasons why the public policy is implemented articulated with constitutional principles and current regulations?

| Totally disagree | Disagree | Neither agree nor disagree | Agree | Totally agree |
| --- | --- | --- | --- | --- |

- Does the design of the policy comply with legally established democratic processes?

| Totally disagree | Disagree | Neither agree nor disagree | Agree | Totally agree |
| --- | --- | --- | --- | --- |

- Were conflicts of interest identified in the formulation and implementation of the policy?

| Yes | No |
| --- | --- |

- Were the conflicts of interest declared in written form?

| Yes | No |
| --- | --- |

- In the event of conflicts of interest, were strategies used to resolve them in accordance with the legal framework and ethical principles?

| Totally disagree | Disagree | Neither agree nor disagree | Agree | Totally agree |
| --- | --- | --- | --- | --- |

- **Participation**

Participation is understood as those exercises in which the institutionality dialogs with citizens to address matters of public interest. In a participatory process, instruments are proposed where the intervention of citizens in the formation, exercise and control of political power is materialized and allowed. The processes of citizen participation allow the formulation of public policies closer and relevant to the territories.

*Questions*

- Was the active participation of people belonging to different social, cultural, or territorial contexts in the design of public policy guaranteed, and were the diverse perspectives found contrasted?

| Totally disagree | Disagree | Neither agree nor disagree | Agree | Totally agree |
| --- | --- | --- | --- | --- |

- Were the results of the participation process included in the design of the public policy?

| Totally disagree | Disagree | Neither agree nor disagree | Agree | Totally agree |
| --- | --- | --- | --- | --- |

- **Equity**

There is equity in public health when there are conditions that allow all people to have good health. To achieve this, the State must promote access to health services and health-promoting environments. In addition, the fact that each person has unique characteristics, and diverse experiences should be recognized, and these must be taken into account to achieve health equity.

*Questions*

- Were the possible inequities in the distribution of benefits and risks of the policy in the target population evaluated?

| Totally disagree | Disagree | Neither agree nor disagree | Agree | Totally agree |
| --- | --- | --- | --- | --- |

- Are strategies envisaged in the public policy to prevent discrimination against vulnerable populations?

| Totally disagree | Disagree | Neither agree nor disagree | Agree | Totally agree |
| --- | --- | --- | --- | --- |

- Are strategies envisaged in the public policy to eliminate discrimination against vulnerable populations?

| Totally disagree | Disagree | Neither agree nor disagree | Agree | Totally agree |
| --- | --- | --- | --- | --- |

- Is there a differential approach based on social determinants and health inequities?

| Totally disagree | Disagree | Neither agree nor disagree | Agree | Totally agree |
| --- | --- | --- | --- | --- |

- **Interculturality**

The concept of interculturality applied to the public sphere allows a critical understanding of how liberal states do not delve into the hierarchical distinction that prevents access to goods and services for certain populations because of their identity, despite recognizing diversity and pluralism. Another critical reason to incorporate an interculturality approach is that it allows us to understand the importance of including new visions that enrich culture and science and reflects on how in the scientific field we should take into account subjects and knowledge that have historically been excluded from this field of knowledge not only as a historical redress but also how this gives openness and reinforces the spirit of scientific knowledge.

*Questions*

- Were cultural and territorial factors of the different communities involved in the design and implementation of the public policy taken into account?

| Totally disagree | Disagree | Neither agree nor disagree | Agree | Totally agree |
| --- | --- | --- | --- | --- |

- Were the conditions of urban, rural, and dispersed territories considered in the design and implementation of the public policy?

| Totally disagree | Disagree | Neither agree nor disagree | Agree | Totally agree |
| --- | --- | --- | --- | --- |

- Does the diagnosis of the policy include an analysis with a differential approach to health inequalities?

| Totally disagree | Disagree | Neither agree nor disagree | Agree | Totally agree |
| --- | --- | --- | --- | --- |

- **Social responsibility**

Social responsibility implies that the efforts to guarantee the promotion of health, prevent diseases and prolong life are not only the responsibility of the public sector, but of a complex network of actors who are connected to commit themselves to guarantee conditions that favor a comprehensive health. This is why private companies, international cooperation, NGOs, the media, among other relevant actors, must join forces to promote citizens' health and comply with certain obligations and regulations so as not to jeopardize the health of the population they influence. In the event that these actors do not comply with certain regulations; the State may intervene to control the situation.

*Questions*

- Were actors identified, both public and private, involved in the problem under the public policy?

| Totally disagree | Disagree | Neither agree nor disagree | Agree | Totally agree |
| --- | --- | --- | --- | --- |

- If incentives directed to the actors are considered to motivate them to reduce their impact on the problem under policy, is there an ethical body evaluating these incentives?

| Yes | No |
| --- | --- |

- Does the public policy establish regulations to mitigate the impact of the actors involved in the problem under policy?

| Totally disagree | Disagree | Neither agree nor disagree | Agree | Totally agree |
| --- | --- | --- | --- | --- |

- **Coercion**

Coercion is defined as the limitation of the autonomy of an individual or a community. A public policy should be as less coercive as possible, however, bearing in mind that public policies for behavioral change often require some coercion, it should be considered that each type of coercion must have a reasonable and sufficient justification. The evaluation of this aspect in this instrument is guided by the scale proposed in the Nuffield Council on Bioethics.

**Coercion scale**

To apply the scale of coercion shown below you must:

- Choose the types of coercion the policy uses and, in each, answer the corresponding question.
- Bearing in mind that the types of coercion are not mutually exclusive, policies generally provide for various types of actions.

| **Types of coercion** | |
| --- | --- |
| The proposed public policy simply monitors the current situation and does not exert any degree of coercion. | Was it assessed whether any degree of restraint is required to address the public policy objective?   - Yes - No |
| The public policy raises educational and informative strategies to face the object of the intervention. | Do educational and informative strategies allow the development of individual autonomy?   - Totally disagree - Disagree - Neither agree nor disagree - Agree - Totally agree |
| The public policy requires people to participate in activities or programs that involve some behavioral change. | Is participation in such activities voluntary?   - Yes - No   Are the activities or programs respectful of human rights?   - Totally disagree - Disagree - Neither agree nor disagree - Agree - Totally agree |
| The proposed public policy guides the elections through behavioral change strategies. | Is there an ethical evaluation of the possible manipulation of behavior derived from the policy and the effects that it may have on the well-being of the person?   - Totally disagree - Disagree - Neither agree nor disagree - Agree - Totally agree |
| The proposed public policy guides the change of behavior of the person through incentives. | Is there an ethical assessment of the relevance of the type and extent of incentive offered?   - Totally disagree - Disagree - Neither agree nor disagree - Agree - Totally agree |
| The proposed public policy guides the change of behavior of the person through disincentives. | Were the proposed disincentives assessed to meet equity criteria?   - Totally disagree - Disagree - Neither agree nor disagree - Agree - Totally agree |
| The proposed public policy partially restricts individual freedoms in order to protect collective health. | Are restrictions on individual freedoms proportionate to the threat to public health?   - Totally disagree - Disagree - Neither agree nor disagree - Agree - Totally agree   Are restrictions within the framework of human rights?   - Totally disagree - Disagree - Neither agree nor disagree - Agree - Totally agree |
| The proposed public policy completely restricts individual freedoms in order to protect collective health. | Are restrictions on individual freedoms proportionate to the threat to public health?   - Totally disagree - Disagree - Neither agree nor disagree - Agree - Totally agree   Are restrictions within the framework of human rights?   - Totally disagree - Disagree - Neither agree nor disagree - Agree - Totally agree   Was the policy evaluated through a bioethics committee or council?   - Yes - No |

.

- **Consent**

People should generally be able to decide for themselves whether to participate in a particular health program after they have been sufficiently informed. Therefore, informed consent is essential whether individual individuals or a whole community are to be intervened. There are times when public health interventions require individual and explicit informed consent, for example, when these involve clinical interventions. But there are other times when community consent is guaranteed through legitimacy in the decision-making process and policy implementation.

*Questions*

- Has the drafting of the consent passed through the evaluation of any ethics committee?

| Yes | No |
| --- | --- |

- Answer this question if the intervention requires individual consent: Did the people targeted by the public policy granted their informed consent to be intervened, if necessary?

| Yes | No |
| --- | --- |

- Does the proposed public policy on behavioral change ensure the protection, privacy and confidentiality of those involved in interventions?

| Totally disagree | Disagree | Neither agree nor disagree | Agree | Totally agree |
| --- | --- | --- | --- | --- |
