# Supplementary material for: Design and validation of a bioethical assessment instrument for public health policies involving behavioral change: A mixed-methods study
Source: Public Health Pract (Oxf). 2026 Feb 9;11:100742. doi: 10.1016/j.puhip.2026.100742 (PMC12915271; doi:10.1016/j.puhip.2026.100742)
Supplement: Multimedia component 2 [file mmc2.docx]

| **Initials** | **Sex** | **Educational level** | **University teacher** | **Employee/Adviser to the Colombian Government** |
| --- | --- | --- | --- | --- |
| AF | F | Master | x | x |
| EF | M | PhD | x | x |
| SLG | F | PhD | x | x |
| RLN | M | PhD |  | x |
| AMG | M | PhD | x | x |
| PM | F | Master |  | x |
| LM | M | Master |  | x |
| MD | F | Master |  | x |
| CO | F | PhD | x | x |
| ER | F | Master | x | x |
| ML | F | PhD | x |  |

Annex 2: Participants Map
