# Supplementary material for: Design and validation of a bioethical assessment instrument for public health policies involving behavioral change: A mixed-methods study
Source: Public Health Pract (Oxf). 2026 Feb 9;11:100742. doi: 10.1016/j.puhip.2026.100742 (PMC12915271; doi:10.1016/j.puhip.2026.100742)
Supplement: Multimedia component 3 [file mmc3.docx]

**Semi-structured interview form**

**General questions**

- What has been your experience in designing or evaluating public health policies?
- Have you participated in the construction or evaluation of public health policies that seek to change the behavior of the population?
- Generally, which are the different actors involved in the development or evaluation of these policies?
- What is your opinion of how public health policies in Colombia are designed or evaluated, particularly those that have a focus on behavioral change?
- Is it possible that ethical conflicts or tensions may arise in this process?
- Has your experience of participating in the design or evaluation of these policies created this type of conflict or tension?
- How have these kinds of ethical conflicts been addressed?
- Is this process usually an ethical reflection on the impact of these policies?
- What criteria should guide these ethical reflections?
- Are there methodologies, instruments, manuals, or criteria to address these ethical conflicts?
- Do you think that such strategies should be implemented?
- Are there any expert committees or teams to guide the ethical analysis of these policies?
- Are there any aspects that have not been addressed in this interview but that you consider relevant to this topic?

**Questions by domain**

- *Transparency:*

1. What elements/aspects of public policies are made known to the target population? At what stage are these aspects made known and how is this information disseminated?
2. What considerations are taken into account when sharing information related to a public policy?
3. What strategies do they use to ensure that public policies are clearly communicated? Are its implications, risks and/or limitations exposed?
4. Is the type of population to which the public policy is directed towards considered in the design of the dissemination of information?

- *Participation*

1. Which people participate in the construction and in which instances? What is their scope of participation?
2. Based on which criteria are participants chosen?
3. What are the effects of participation in the construction of public policies? Has it limited its application? Has it led to the improvement of public policy?

- *Evidence*

1. What role does scientific evidence play in the process of public policy-making?
2. How is the evidence evaluated when building public policy?
3. Who is responsible for seeking evidence for the implementation of public policy? What is that search process like?
4. What strategies are used when there is no evidence?

- *Economic evaluation*

1. What criteria are considered when funding a particular public policy?
2. What are the criteria for investing in a public policy?
