# Supplementary material for: Design and validation of a bioethical assessment instrument for public health policies involving behavioral change: A mixed-methods study
Source: Public Health Pract (Oxf). 2026 Feb 9;11:100742. doi: 10.1016/j.puhip.2026.100742 (PMC12915271; doi:10.1016/j.puhip.2026.100742)
Supplement: Multimedia component 4 [file mmc4.docx]

### ****Appendix 4****

A descriptive study with an exploratory, qualitative design was conducted to examine participants’ understanding and perceptions through semi-structured interviews. In addition, the study followed a phenomenological approach aimed at exploring how participants experienced and interpreted these issues within their own frames of reference, without attempting to establish causal relationships. Participants were intentionally selected based on a previously constructed map that included local and national experts. The sampling was one of convenience, and the final number of participants depended on the saturation of emerging categories.

Interviews were recorded by one or two members of the research team after participants signed informed consent. All recordings were transcribed, and the textual data were imported into ATLAS.ti for analysis.

### ****Results****

A total of 10 interviews were conducted, six men and four women, from different sectors, particularly academia and governmental institutions, including Ministries, Health Secretariats, and Congress.

A thematic analysis was performed to explore and interpret the textual data, guided by the research question and the Colombian and Latin American context. The analysis followed the six steps described by Braun and Clarke, as referenced in the article. One researcher conducted the initial analysis, beginning with data familiarization, followed by inductive coding. From this process, the following themes were developed:

1. Legitimacy
2. Participation
3. Intersectoral collaboration
4. Evidence
5. Equity
6. Challenges and opportunities in using an instrument to evaluate the bioethical dimension of public policies

A report was then drafted, selecting representative quotations that clearly illustrated each theme. The report was subsequently discussed and refined with the other researchers.

Interviewees had participated in various public policies involving behavior-change components, such as healthy eating, sexual and reproductive health, gender-based violence, asbestos-use restriction, and vaccination. One participant was a member of the recently established National Bioethics Committee, created as an advisory body.

A central finding was that interviewees unanimously indicated they were not aware of any instrument designed to assess the bioethical aspects of public policies. Only one participant questioned whether such a tool is the appropriate approach for evaluating or designing public policies.

**Legitimacy** emerged as the first category, including the subcategories of evidence, participation, and intersectorality. However, most references within this category pointed to conflicts of interest experienced by interviewees in these policy processes.

**Participation** was emphasized as essential in policy design. Several difficulties were identified, including misalignment in stakeholders’ perspectives, fractures in dialogue, asymmetries in knowledge of the process, lack of commitment from institutional actors, particularly in the regions, insufficient stakeholder engagement, and inadequate discussion.

**Intersectorality** was described as essential and inherently diverse, especially in the design of public policies.

Regarding **evidence**, interviewees expressed varying and sometimes contradictory views. Some noted a lack of evidence in the process, particularly research not conducted in Colombia, while others mentioned that evidence often becomes the dominant criterion, overshadowing equally or more relevant considerations. A recurrent concern was that evidence is not always the starting point. Initiatives may originate with political or other interests, creating tension between political will and evidence.

The second major category, **Equity**, emerged both as a requirement and as a challenge. It was associated with justice and the exercise of power, particularly by the State, applied in differential ways, reflecting intersectional disparities, and influenced by interests and political agendas.

Regarding the feasibility of developing an instrument to assess the bioethical aspects of public policies, conflicts of interest were again highlighted as potential barriers to its application and usefulness. Another challenge was the influence of the political agenda, which consists of motivations that guide policy processes and may act as either a facilitator or an obstacle.

In terms of **policy evaluation**, participants noted that the evaluation process is defined by the National Planning Department and must be established from the design stage. This evaluation follows predefined norms linked to a value chain and policy objectives but does not incorporate a bioethical perspective. Interviewees proposed broader perspectives that would integrate elements not currently included in regulations and involve actors not traditionally considered in policy evaluation.
