# Supplementary material for: Design and validation of a bioethical assessment instrument for public health policies involving behavioral change: A mixed-methods study
Source: Public Health Pract (Oxf). 2026 Feb 9;11:100742. doi: 10.1016/j.puhip.2026.100742 (PMC12915271; doi:10.1016/j.puhip.2026.100742)
Supplement: Multimedia component 5 [file mmc5.docx]

**Delphi Methodology: Instrument for bioethical evaluation of public health policies with a behavioral change component**

Thanks for participating in the first feedback of the Instrument for Bioethical Assessment of Public Health Policies with a Behavioral Change Component. The Bioethics Center gathered your suggestions and from 11 other experts and integrated them into the development of a second version of the instrument.

We are now inviting you for a second evaluation. The form has the same guiding questions as the first feedback, but the purpose is now to give feedback about this second version of the instrument.

Remember that the instrument you have been evaluating is intended to assess the bioethical dimension of public health policies with a behavioral change component, based on theoretical foundations such as the Tools and Ethics for Applied Behavioral Insights: The BASIC Toolkit (OECD) and the management model proposed by the Nuffield Council on Bioethics.

Only the professionals involved in building the instrument (researchers) know that you are participating in this feedback exercise. If the results of this feedback exercise are published, you will not be identified by name.

1. Names and Surnames

**Closed Questions**

Answer by giving a score of 1 to 7 with 1 being completely in agreement, 2 strongly in agreement, 3 in agreement, 4 neutral, 5 in disagreement, 6 strongly in disagreement, and 7 completely in disagreement.

1. Does the design and wording of the questions convey the information intended to be obtained in accordance with the objectives of the research?

| 1 | 2 | 3 | 4 | 5 | 6 | 7 |
| --- | --- | --- | --- | --- | --- | --- |

1. Are the questions organized according to the established domain structure?

| 1 | 2 | 3 | 4 | 5 | 6 | 7 |
| --- | --- | --- | --- | --- | --- | --- |

1. Does the wording of the questions make them easy to understand?

| 1 | 2 | 3 | 4 | 5 | 6 | 7 |
| --- | --- | --- | --- | --- | --- | --- |

1. Are there any repetitive questions?

| 1 | 2 | 3 | 4 | 5 | 6 | 7 |
| --- | --- | --- | --- | --- | --- | --- |

1. Are there any repetitive questions?

| 1 | 2 | 3 | 4 | 5 | 6 | 7 |
| --- | --- | --- | --- | --- | --- | --- |

1. Do the questions have an overly technical language?

| 1 | 2 | 3 | 4 | 5 | 6 | 7 |
| --- | --- | --- | --- | --- | --- | --- |

1. Should one or more questions be added?

| 1 | 2 | 3 | 4 | 5 | 6 | 7 |
| --- | --- | --- | --- | --- | --- | --- |

1. Do you think one or more questions need to be removed?

| 1 | 2 | 3 | 4 | 5 | 6 | 7 |
| --- | --- | --- | --- | --- | --- | --- |

1. With regard to the scale used in the section on coercion, is it appropriate for the information to be obtained?

| 1 | 2 | 3 | 4 | 5 | 6 | 7 |
| --- | --- | --- | --- | --- | --- | --- |

1. Is the instrument very extensive?

| 1 | 2 | 3 | 4 | 5 | 6 | 7 |
| --- | --- | --- | --- | --- | --- | --- |

1. Are there any necessary issues that are not covered by this instrument?

| 1 | 2 | 3 | 4 | 5 | 6 | 7 |
| --- | --- | --- | --- | --- | --- | --- |

**Open-ended questions**

Answer the following questions freely and openly. After each question there is a space for you to answer with the extension you think necessary

1. What is your opinion of the wording and order of the questions in the instrument?
2. Would you modify any questions? What question would you modify and how?
3. Would you add or remove any questions? Which one and why?
4. What is your opinion of the instrument in general?
5. At what point in the development of a public policy would it be relevant to implement this instrument?

Thank you so much!
